# Endocrine disrupting chemicals: A promoter of non-alcoholic fatty liver disease

**DOI:** 10.3389/fpubh.2023.1154837

**Published:** 2023-03-22

**Authors:** Yajie Chen, Yang Wang, Ziqiang Cui, Wenpeng Liu, Baowang Liu, Qiang Zeng, Xin Zhao, Jian Dou, Jinglin Cao

**Affiliations:** Department of Hepatobiliary Surgery, Third Hospital of Hebei Medical University, Shijiazhuang, Hebei, China

**Keywords:** non-alcoholic fatty liver disease, endocrine-disrupting chemicals, per-/polyfluorinated substance, bisphenol A, polychlorinated biphenyls, phthalates

## Abstract

Non-alcoholic fatty liver disease (NAFLD) is the most prevalent liver disorder. With the improvement in human living standards, the prevalence of NAFLD has been increasing in recent years. Endocrine-disrupting chemicals (EDCs) are a class of exogenous chemicals that simulate the effects of hormones in the body. There has been growing evidence regarding the potential effects of EDCs on liver health, especially in NAFLD. This paper aims to summarize the major EDCs that contribute to the growing burden of NAFLD and to raise public awareness regarding the hazards posed by EDCs with the objective of reducing the incidence of NAFLD.

## 1. Introduction

Non-alcoholic fatty liver disease (NAFLD) is an important public health issue that affects a large portion of the global population. Estimates indicate that the worldwide prevalence of NAFLD ranges from 13% in Africa to 42% in Southeast Asia (1). NAFLD encompasses a spectrum of liver conditions, including simple steatosis or non-alcoholic fatty liver (NAFL), which has a milder course, and non-alcoholic steatohepatitis (NASH), with potential progression to fibrosis or cirrhosis and hepatocellular carcinoma (HCC) (2, 3). The hallmark of NAFLD is the accumulation and deposition of excessive fat in liver cells, which may be related to genetic, dietary, and environmental factors (4). These factors promote the onset of insulin resistance (IR) in adipose tissue, leading to adipocyte dysfunction and increased influx of free fatty acids (FFAs) into the liver (5). These FFAs and their consequent lipotoxic intermediates have been shown to have adverse effects such as abnormal lipids metabolism, oxidative stress, and chronic liver inflammation, all of which contribute to the progression of NAFLD (6, 7).

Environmental endocrine disrupting chemicals (EDCs) are a class of exogenous chemicals that mimic the effects of hormones in the body, causing hormonal dysregulation and mediating various metabolic disorders. The liver, an organ crucial to metabolism and detoxification (8), has been shown to be impacted by EDCs, with studies suggesting exposure to these chemicals can lead to metabolic changes and liver disease (9–12). Because of the difficulty in getting biopsy-confirmed NAFLD histological specimens, the liver injury is typically assessed using serum biomarkers of hepatoxicity of NAFLD. Alanine aminotransferase (ALT), aspartate aminotransferase (AST), alkaline phosphatase (ALP) and γ-glutamyl transferase (GGT) are considered specific biomarkers of liver injury, they are widely used to evaluate the progression of NAFLD. Given the role of EDCs in the worldwide deterioration of metabolic health, it is imperative that the scientific community continues to study and understand their effects. We aimed to provide an overview of EDCs and summarize the effects of EDC exposure on NAFLD.

### 1.1. Overview of EDCs

Environmental endocrine disruptors (EDCs) are a heterogenous group of chemicals that are widely distributed and easily enriched, present in many forms. In daily life, an increasing number of substances have been identified as EDCs; they enter the body through the digestive tract, respiratory tract, or skin, and produce adverse effects. EDCs can be classified as natural or synthetic, based on their origin. Natural EDC include phytoestrogens and mycotoxins, while synthetic EDCs include chemicals used as industrial solvents and their byproducts (polychlorinated biphenyls, polybrominated biphenyls, and dioxins), plastics (bisphenol A), plasticizers (phthalates), fungicides (vinclozolin), pesticides (methoxychlor and chlorpyrifos), heavy metals (mercury and lead), and pharmaceutical agents present in human and animal foods (11, 13–16).

Studies have shown that EDCs can interfere with various aspects of hormone regulation in the body, including production, release, transport, metabolism, binding, action, and elimination, leading to hormonal dysregulation and contributing to various metabolic disorders (17, 18). It’s also been found that EDCs play a potential role in the regulation of genomic expression, promoting epigenetic modifications that result in the development of pathologies by mediating carcinogenic, neurotoxic, hepatotoxic, and immunotoxic effects (19–21). The challenge in understanding the impact of EDCs is compounded by the fact that humans are exposed, not to a single environmental pollution compound, but to a cocktail of EDCs, making it even more difficult to predict the net effect and evincing the association between a specific EDC and disease (13, 22). Moreover, exposure to persistent EDCs may initiate and promote the pathogenesis of NAFLD (23). EDCs affect the progression of NAFLD through the interaction of nuclear receptors. Activating transcription factors, triggering the imbalances between lipid flow/outflow in the liver, promoting mitochondrial dysfunction, and mediating the hepatic inflammatory are the possible mechanism of NAFLD (17).

## 2. Methods

In this study, we focused on several key EDCs that are closely related to human health, including per−/polyfluorinated substances, bisphenol A, polychlorinated biphenyls, and phthalates (as outlined in Table 1). Systematic search of PubMed and Embase databases was conducted from January 1, 2010, to December 28, 2022, to identify human studies investigating the relationship between non-alcoholic liver disease and these EDCs. Detailed search strategies are presented in Table 2. After screening the retrieved studies based on their titles and abstracts, we excluded studies without human data, case reports, non-original reports, studies without NAFLD outcomes, and pharmacological or ecological studies.

**Table 1:** Characteristic of major endocrine-disrupting chemicals (EDCs).

Substance	Abbreviation	Source	Characteristic
Per-/polyfluorinated substance	PFAS	Contaminated drinking water, foods, air, etc	Environmental persistence, bioaccumulation, potential hazards
Polychlorinated Biphenyls	PCBs	Electrical equipment, soil, aquatic sediments, contaminated food, etc	Thermodynamically stable, degradation-resistant, bioaccumulation
Bisphenol A	BPA	Plastic containers and toys, food packaging materials, dental sealants, etc	Low lipophilicity, rapid degradation, short half-time
Phthalates	DEPH	Plasticizers in food wrapping and packaging, coatings, cosmetics, adhesives, medical tubes, ect	Rapid metabolism, strong adsorption

**Table 2:** Literature review search terms.

Substances	Database	Search terms
NAFLD	PubMed	NAFLD OR NASH OR “nonalcoholic fatty liver disease” OR “nonalcoholic steatohepatitis” OR “nonalcoholic fatty liver” OR “fatty liver” OR steatosis OR “liver enzymes” OR“liver damage” OR “liver injury” OR “liver fibrosis”
	Embase	nafld OR nash OR ‘nonalcoholic fatty liver disease’/exp. OR ‘nonalcoholic fatty liver disease’ OR ‘nonalcoholic steatohepatitis’/exp. OR ‘nonalcoholic steatohepatitis’ OR ‘nonalcoholic fatty liver’/exp. OR ‘nonalcoholic fatty liver’ OR ‘fatty liver’/exp. OR ‘fatty liver’ OR ‘steatosis’/exp. OR steatosis OR ‘liver enzymes’/exp. OR ‘liver enzymes’ OR ‘liver damage’/exp. OR ‘liver damage’ OR ‘liver injury’/exp. OR ‘liver injury’ OR ‘liver fibrosis’/exp. OR ‘liver fibrosis’
Per-/polyfluorinated substance	PubMed	Perfluoroalkyl OR Polyfluoroalkyl OR Perfluorinated OR polyfluorinated OR perfluoro* OR polyfluoro* OR PFAS OR PFAS* OR “Perfluorinated chemicals” OR Perfluorocarbons OR Polyfluorocarbons OR “Per- and Polyfluoroalkyl Substances”
	Embase	‘perfluoroalkyl’/exp. OR perfluoroalkyl OR ‘polyfluoroalkyl’/exp. OR polyfluoroalkyl OR perfluorinated OR polyfluorinated OR perfluoro* OR polyfluoro* OR pfas OR pfas* OR ‘perfluorinated chemicals’ OR perfluorocarbons OR polyfluorocarbons OR ‘per- and polyfluoroalkyl substances’
Bisphenol A	PubMed	BPA OR “Bisphenol A” OR Bisphenol* OR “bisphenol A glycidyl methacrylate” OR “4,4-dihydroxy-2,2-diphenylpropane” OR “diphenylolpropane” OR “2,2-bis(4-hydroxyphenyl)propane” OR ‘bisphenol A, sodium salt” OR “bisphenol A, disodium salt”
	Embase	bpa OR ‘bisphenol a’ OR bisphenol* OR ‘bisphenol a glycidyl methacrylate’ OR ‘4,4-dihydroxy-2,2-diphenylpropane’ OR ‘diphenylolpropane’ OR ‘2,2-bis(4-hydroxyphenyl)propane’ OR ‘bisphenol a, sodium salt’ OR ‘bisphenol a, disodium salt’
Polychlorinated Biphenyls	PubMed	PCB OR PCB* OR “Polychlorinated Biphenyls” OR “Polychlorobiphenyl Compounds” OR “Polychlorinated Biphenyl” OR PBB OR PBB* or “Polybrominated biphenyls” OR “Polybromobiphenyl Compounds” OR “Polychlorinated terphenyls” OR PCN OR PCN* OR “Polychlorinated naphthalenes”
	Embase	‘pcb’/exp. OR pcb OR pcb* OR ‘polychlorinated biphenyls’/exp. OR ‘polychlorinated biphenyls’ OR ‘polychlorobiphenyl compounds’ OR ‘polychlorinated biphenyl’/exp. OR ‘polychlorinated biphenyl’ OR pbb OR pbb* OR ‘polybrominated biphenyls’/exp. OR ‘polybrominated biphenyls’ OR ‘polybromobiphenyl compounds’ OR ‘polychlorinated terphenyls’ OR pcn OR pcn* OR ‘polychlorinated naphthalenes’
Phthalates	PubMed	“di-2-ethylhexyl phthalate” OR phthalate OR DEHP OR “Di(2-ethylhexyl) phthalate” OR “Phthalate” OR “Phthalates” OR “Dibutyl phthalate” OR “di-n-butyl phthalate” OR “di-isobutyl phthalate” OR DBP OR DiBP OR “mono (2-ethylhexyl) phthalate” OR “MEHP” OR “monomethyl phthalate” OR “mono (2-ethyl-5-carboxypentyl) phthalate” OR “MBP” OR “mono-(3-carboxypropyl) phthalate”
	Embase	‘di-2-ethylhexyl phthalate’/exp. OR ‘di-2-ethylhexyl phthalate’ OR phthalate OR dehp OR ‘di(2-ethylhexyl) phthalate’/exp. OR ‘di(2-ethylhexyl) phthalate’ OR ‘phthalate’/exp. OR ‘phthalate’ OR ‘phthalates’ OR ‘dibutyl phthalate’/exp. OR ‘dibutyl phthalate’ OR ‘di-n-butyl phthalate’/exp. OR ‘di-n-butyl phthalate’ OR ‘di-isobutyl phthalate’ OR ‘dbp’/exp. OR dbp OR dibp OR ‘mono (2-ethylhexyl) phthalate’/exp. OR ‘mono (2-ethylhexyl) phthalate’ OR ‘mehp’ OR ‘monomethyl phthalate’/exp. OR ‘monomethyl phthalate’ OR ‘mono (2-ethyl-5-carboxypentyl) phthalate’ OR ‘mbp’ OR ‘mono-(3-carboxypropyl) phthalate’

## 3. Relationship between NAFLD and EDCs

### 3.1. Per-/polyfluorinated substance

Per-/polyfluorinated substance (PFAS) is a series of organic compounds containing at least one perfluorinated carbon atom, it is lipophobic and hydrophobic that are useful for manufacturing wide ranges of consumer products (24, 25). PFASs have a stable chemical structure with a half-life of about 2–8 years, allowing them to persist and accumulate in the environment (10, 26). Because of these properties, PFASs are classified as “persistent organic pollutants” (POPs), and their delayed-elimination feature may cause long-term harmful health effects. PFASs have been detected in drinking water, various foods (meat, vegetables, milk, eggs), air, and early life placental or breast milk, and are able to accumulate in biological tissues and organs with high protein content (27–29). Nearly all adults in the U.S. have been found to have accumulated PFAS in their body tissues (10). Current studies have indicated that four congeners account for most human exposure: perfluorooctanesulfonic acid (PFOS), perfluorooctanoic acid (PFOA), perfluorohexanesulfonic acid (PFHxS), and perfluorononanoic acid (PFNA) (10, 30, 31). Compared to the general population, worker in certain occupations (e.g., professional ski-waxers, firefighters, fluorochemical plant workers) experience high PFAS serum concentrations based on their occupation (32). The median concentrations were 24-27 ng/ml (PFOS), 50-57 ng/ml (PFOA), 1.4–1.6 ng/ml (PFHxS) and 12-13 ng/ml (PFNA) in professional ski-waxers (33). And the median concentrations in the general population were 3.59–24.22 ng/mL (PFOS) (34, 35), 0.99–28.0 ng/mL (PFOA) (36, 37), 0.59–1.80 ng/mL (PFHxS) (38, 39), and 0.24–1.60 ng/mL (PFNA) (34, 37), respectively.

The liver is one of the main target organs of PFAS toxicity (40). However, the exact mechanism of PFAS hepatotoxicity remains unclear. PFAS are thought to act as ligands for peroxisome proliferator-activated receptors (PPARs), which promote liver inflammation and triglyceride accumulation through the activation of PPARα and lead to liver injury or NAFLD (41–43). The complementary mechanism also includes activation of the constitutive androstane receptor (CAR) (42, 44), downregulation of nuclear factor erythroid 2-related factor 2 (NRF2) (45, 46), and upregulation of nuclear factor kappa-light-chain-enhancer of activated B cells nuclear factor-kappa B (NF-ĸ B) (47). Animal and epidemiological studies have shown that PFAS can cause an obviously increase in liver lipid volume, induce mitochondrial dysfunction and oxidative stress, and promote inflammatory responses in NAFLD progression (41, 48, 49).

A large number of nuclear receptors (NRs) were expressed in liver, making it a critical target for PFAS. In this review, we noticed that a few studies have evaluated the hepatic enzyme abnormalities associated with PFAS exposure. PFOA and PFNA exposure is usually positively associated with higher ALT and GGT levels, suggesting that changes in serum biomarkers are often accompanied by histopathological changes or liver disease (34, 39, 50–53). However, for AST, studies have reported different outcomes in different crowds. Khalil (37) found that there was no relationship between serum PFAS and AST levels in obese children, while PFOA and PFOS were positively correlated with AST in Japanese children (52). In addition, Attanasio et al. reported sex-specific histological effects of PFAS exposure and found positive associations between PFAS and ALT in female adolescents, but conversely in male adolescents, which could be mediated by sex hormones (54). Evidence for sex-specific differences was also found in rats, with ALT increasing more frequently in male rats (55–57). The authors suggest that PFAS exposure may play an important role in the development of NAFLD and carcinoma (Table 3). The PFAS was positively associated with lobular inflammation in adults undergoing bariatric surgery in Northern Europe, however, the reason for this association is unclear. It may be related to changes in lipid and bile acid metabolism (9, 59). In animal models, exposure to subchronic PFOS has been found to enhance hepatic stellate cell (HSC) activation and exacerbate carbon tetrachloride (CCl_4_)-induced liver fibrosis (60), consistent with the outcomes of Sen’s study, which showed that PFOS was positively associated with hepatic fibrosis in adults (9). In contrast, a study including adults from the C8 Health Project in the USA showed that cumulative PFOA exposure had no effect on all liver diseases, enlarged liver, or cirrhosis (58). Additionally, a study included 1,105 mother–child pairs from the European Human Early-Life Exposome (HELIX) cohort showed that higher exposure to PFAS during pregnancy was associated with higher liver enzyme levels in children (38). PFAS can cross the placenta barrier efficiently and deposit in fetal tissues (61), they altered some amino acid (e.g., valine, leucine, phenylalanine) and lipid (e.g., glycerophospholipid) metabolism that related to NAFLD pathogenesis, which exerted adverse effects in liver (38).

**Table 3:** Epidemiologic studies on the relationship between PFAS and NAFLD or carcinoma.

Substance	Reference	Country	Population	Sample size	Biological materials	Measurement	Exposure assessment	Outcomes	Results	Adjustment factors
PFAS	Sen et al. (9)	Sweden	Adults undergoing laparoscopic bariatric surgery (18–75 years)	105	Serum	UPLC-QTOFMS	PFHxS, PFNA, PFOA, PFOS	NASH, hepatic fibrosis, macrosteatosis	PFOS, PFOA were positively associated with NASH (necroinflammatory grades), while PFOS was positively associated with hepatic fibrosis	NR
PFAS	Jin et al. (35)	USA 2007–2015	Children with NAFLD	74	Plasma	HRMS	ORs and 95%CI for liver histology in relation to PFOA, PFOS, PFHxS, PFAS score (per IQR increase)	Histologic severity of NAFLD	The odds of having NAFLD was significantly increased with each IQR increase of PFOS and PFHxS. Each IQR increase of PFHxS was associated with increased OR for liver fibrosis, lobular inflammation and higher NAFLD activity score.	NR
PFAS	Darrow et al. (58)	USA 2008–2011	C8 Health Project (age ≥ 20 years)	28,047	Serum	Exposure estimation	Median PFOA: 1.65 ng/mL	Validated liver disease, medically validated enlarged liver, fatty liver, cirrhosis	No evidence of an effect of cumulative PFOA exposure on all liver disease, nor on enlarged liver, fatty liver, and cirrhosis.	Age, sex, BMI, alcohol consumption, race, regular exercise, smoking status, education, household income, fasting status, worker at plant, insulin resistance
PFAS	Rantakokko et al. (59)	Finland 2005–2010	Kuopio Obesity Surgery Study	161	Serum	NR	Median(5th, 95th) ng/mL PFOA: 2.56(1.04, 4.66) PFNA: 0.83(0.30, 2.19) PFOS: 3.2(0.89–10.3) PFHxS: 1.18(0.54–2.90)	Steatosis, lobel inflammation, ballooning, fibrosis, liver phenotype	PFOA, PFNA, and PFHxS were inversely associated with lobular inflammation at baseline.	Age, fasting insulin, weight change

HRMS, high-resolution mass spectrometry; LC-HRMS, liquid chromatography with high-resolution mass spectrometry; UPLC-QTOFMS, ultra-high-performance liquid chromatography quadrupole time-of-flight mass spectrometry.

### 3.2. Polychlorinated biphenyls

Polychlorinated biphenyls (PCBs) are also a type of POP that are manufactured and used commercially as dielectric fluids in transformers (62). They are thermodynamically stable polyhalogenated aromatic hydrocarbons consisting of up to ten chlorine substituents attached to a biphenyl ring (63). Based on their structures, PCB congeners have been subclassified into dioxin-like (DL) and nondioxin-like (NDL). DL PCBs have a coplanar structure, whereas NDL PCBs have a noncoplanar structure, which can be attributed to receptor-based modes of action. PCBs persist in the environment and accumulate in soil, aquatic sediments, and in species that consume these sources (fish, cows, dairy) (64). Despite a ban on their production and emission in 1979, human exposure to PCBs usually occurs through contaminated air, water, or food. As POPs, PCBs accumulate in adipose tissue and are gradually released into the bloodstream (65, 66).

The liver appears to be both a target and an effector organ for PCB-induced endocrine disruption. The occurrence of NAFLD is due to an imbalance between lipid production and elimination, which promotes excessive accumulation of hepatic lipids (17). PCBs have been documented to be related to this phenomenon, as they can induce pathological fat aggregation, both in the DL and NDL groups (67). DLPCBs activate the aryl hydrocarbon receptor (AhR) and peroxisome proliferator-activated receptors alpha and gamma (PPARα/γ) (63, 68), exerting a multimodal effect on lipid accumulation and causing steatosis by disrupting liver lipid metabolism. NDL PCBs, on the other hand, activate the constitutive androstane receptor (CAR) and pregnane X receptor (PXR) (69), which could reduce the protective response of the liver to promote diet induced NAFLD (67).

Studies have shown that PBC exposure can cause NAFLD related metabolic disorders, including insulin resistance, obesity, and lipid metabolic dysfunction. PCBs differentially regulate hepatic lipid metabolism and several related genes. PCB126 exposure increases hepatic lipids and causes toxicant-associated steatosis (mild small-droplet macro vesicular steatosis) (63). Mono-exposure to either PCB126 or Aroclor1260 increased hepatic lipid uptake while decreasing lipid biosynthesis, but these effects were abrogated by exposure to the NDL/DL PCB mixture (63). Pnpla3, a lipase implicated in NAFLD (70), was significantly upregulated by Aroclor1260 exposure, but was suppressed by either PCB126 or Aroclor1260/PCB126 exposure, potentially due to activation of different receptors among NDL or DL PCBs. PCBs have also been associated with the promotion of toxicant-associated steatohepatitis (TASH), which can ultimately lead to secondary liver necrosis, potentially due to a loss of protein phosphorylation and downregulation of the hepatic kinome (71). Positive associations were also observed between PCB concentrations and NAFLD-related biomarkers (Table 4), with most PCB congeners positively correlated with elevated alanine aminotransferase (ALT) levels. A study of 4,582 adults conducted by Cave et al. (76) showed that 10.6% of participants had unexplained ALT elevation, with older age significantly associated with total PCB levels in the highest quartile. Of those participants aged 70 years or older, 71.7% had high PCB levels compared to only 2.2% of those aged under 30 years. A study of 1,108 mother–child pairs from six countries by Midya (72) discovered that prenatal exposure to PCBs is a potential risk factor for pediatric NAFLD, and were further associated with increased CK-18 levels (a novel marker of hepatocyte apoptosis and NAFLD). Notably, researchers have identified potential therapeutic targets for improving PCB-induced NAFLD, including the anti-fibrotic compound recombinant FGF21, which reduced the overexpression of hepatic lipocalin-2 (LCN2), a group of transporters of small lipophilic molecules that are upregulated in several liver diseases, and attenuated NAFLD (62, 77).

**Table 4:** Epidemiologic studies on the relationship between PCBs and NAFLD related biomarkers.

Substance	Reference	Country	Population	Sample size	Biological materials	Measurement	Exposure assessment	Outcomes	Results	Adjustment factors
PCB	Midya et al. (72)	France, Greece, Lithuania, Norway, Spain, UK 2021–2022	Mother–child pairs from the Human Early-Life Exposome project	1,108	Serum	GC-MS/MS	LOD used in NIPH PCB180: 0.91 pg./g PCB170: 0.61 pg./g PCB153: 0.61 pg./g PCB138: 0.61 pg./g PCB118: 0.31 pg./g	ALT, AST, GGT and CK-18 of children	A 1-quartile increase in prenatal exposure was associated with increased CK-18 for PCBs and constitute a potential risk factor for pediatric non-alcoholic fatty liver disease.	Subcohort, maternal age, maternal prepregnancy BMI, maternal educational level, parity, child age, child sex
PCB	Rantakokko et al. (59)	Finland 2005–2010	Kuopio Obesity Surgery Study	161	Serum	NR	Median ng/g lipid PCB-118: 15.2 (normal) 9.42(steatosis) 10.1(NASH)	Steatosis, lobel inflammation, ballooning, fibrosis, liver phenotype	PCB-118 was associated with NASH, lobular inflammation, few liver cell balloon, and S2-S3 steatosis grade at baseline.	Age, BMI, sex, fasting insulin
PCB	Clair et al. (73)	USA	ACHS adults	738	Serum	HRGC/HRMS	PCB congeners (28, 44, 49, 52, 66, 101, 105, 110, 128, 149, 151, 172, 178, 187, 195)	TASH, CK18	TASH was associated with increased exposures to specific PCB congeners.	Age, BMI, gender, race, diabetes status, alcohol use, total lipid levels
PCB	Kumar et al. (74)	Sweden	Prospective Investigation of the Vasculature in Uppsala Seniors (≥70 years)	992	Serum	HRGC–MS	PCB congeners (74, 99, 105, 118, 126, 138, 153, 156, 157, 169, 170, 180, 189, 194, 206, 209)	Bilirubin, ALP, ALT, GGT	PCBs was not associated with bilirubin, ALP, and GGT. PCB-74, 105, and 118 were found to be significant in positive direction with ALT.	Age, sex, kidney function, smoking BMI, education, physcial activity, waist circumference, fasting blood glucose, systolic blood pressure, use of cardiovascular medication
PCB	Serdar et al. (65)	USA 2003–2004	NHANES (>12 years)	1,935	Serum	HRGC/HRMS	PCB congeners	ALT, AST, GGT	Liver enzymes (AST, ALT, GGT) were significantly higher in the highest exposure groups of PCBs. ALP dropped as levels of PCBs increased.	Age, gender, relevant survey design, subsample, population weights
PCB	Christensen et al. (75)	USA 2003–2004	NHANES (>12 years)	1,345	Serum	HRGC/HRMS	PCB congeners (DL and NDL)	ALT	The DL PCB, the NDL PCB were significant associated with elevated ALT.	Age, sex, race/ethnicity, income, BMI
PCB	Cave et al. (76)	USA 2003–2004	NHANES adults	4,582	Serum	HRGC/HRMS	PCB congeners	ALT	9 of coplanar PCBs (66, 74, 105, 118, 126, 156, 157, 167, 169) were positively associated with elevated ALT. 11 of NDL PCBs (138 and 158, 146, 151, 153, 170, 172, 177, 178, 183, 187, 196 and 203,) were positively associated with ALT elevation.	Age, race/ethnicity, sex, BMI, poverty income ratio, insulin resistance.

GC-MS, gas chromatograph-mass spectrometry; GC-MS/MS, gas chromatography coupled to tandem mass spectrometry; HRGC/HRMS, high-resolution gas chromatography/isotope dilution high-resolution mass spectrometry; HRGC-MS, high-resolution gas chromatograph coupled to mass spectrometry.

### 3.3. Bisphenol A

Bisphenol A (BPA), which consists of two phenol rings attached by a methyl bridge with two methyl groups (78), is a plasticizer mainly used for polycarbonate plastics and epoxy resins in many consumer products (79). BPA exposure can occur *via* various sources such as plastic containers, toys, water bottles, food packaging materials, office supplies, and dental sealants (80, 81). BPA has low lipophilicity and degrades rapidly with a half-life of 4–5 h (82). Due to its broad application, BPA is detected in more than 90% of people, and the median urine BPA concentration in adults is 2.24–6.17 ng/mL (83, 84).

The liver is the main organ that metabolizes and transforms BPA into glucuronidation; therefore, it is more susceptible to BPA than other organs (85). BPA increases the risk of NAFLD owing to fat accumulation, obesity, and oxidative stress. Upregulation of lipogenic enzymes and transcription factors, such as sterol regulatory element binding protein-1c (*srebp-1c*), the carbohydrate responsive element binding protein (ChREBP), and liver X receptor (LXR) (86), promotes *de novo* lipogenesis (DNL) (87), increasing the risk of lipid accumulation and obesity. In addition, exposure to high doses of BPA decreased the activities of antioxidant indicators, such as superoxide dismutase (SOD) and glutathione (GSH), causing excessive accumulation of free radicals, such as reactive oxygen species (ROS), promoting liver damage and hepatotoxicity (88).

Aminotransferases are the most widely used biomarkers in experiments, and are released into the bloodstream following liver injury (89). Epidemiological studies have shown that BPA exposure could have negative effects on NAFLD-related biomarkers (Table 5). Higher urinary BPA levels usually led to the elevation of ALT (83, 96, 97). A study carried out by Lang et al. reported that higher BPA levels are linked to abnormal GGT (Odds ratio [OR]:1.29, 95% Confidence Interval [CI]:1.14–1.46) and ALP (OR:1.48, 95%CI:1.18–1.85) (98). In addition to liver biomarkers, urinary BPA levels were positively associated with the prevalence of NAFLD in adults and adolescents. In the Korean National Environmental Health Survey (83), which included 3,476 participants with a mean age of 52.96 ± 0.25 years old, the geometric mean concentration of BPA in the NAFLD group was significantly higher than in the non-NAFLD group (2.56 ug/L vs. 2.24 ug/L, *p* = 0.001). A study analyzing adolescents (12–19 years old) from NHANES in the USA also showed that the risk of suspected NAFLD (ALT≥30 U/L) was increased in participants in higher quartiles of BPA exposure (93). In terms of pathological findings, mice and rats treated with BPA showed liver tissue dilatation of sinusoids, congestion, inflammation, and necrosis in a dose-dependent manner (99). Although BPA can be excreted quickly from the body, people are constantly exposed to it throughout their lives and BPA exposure is associated with metabolic health in offspring. Prenatal BPA exposure has been shown to alter gene expression profiles and result in peripheral insulin resistance and liver lipotoxicity (100, 101). Gestational BPA exposure can promote the development of NAFLD in rodent models through the perturbation of the nuclear transcription factor activity (102). Another study indicated that exposure to BPA may diminish the immune response following hepatitis B vaccination (79).

**Table 5:** Epidemiologic studies on the relationship between BPA and NAFLD related biomarkers.

Substance	Reference	Country	Population	Sample size	Biological materials	Measurement	Exposure assessment	Outcomes	Results	Adjustment factors
BPA	Fu et al. (90)	China 2017–2018	Children (5–14 years)	1,006	Serum	HPLC	Median BPA: 26.31 ng/mL	ALT, AST, TBIL	Exposure to BPA would have negative effects on hepatic function, and these effects showed differences in gender and geographical location.	Age, address, gender
BPA	An et al. (83)	Korea 2015–2017	KoNEHS (≥18 years)	3,476	Urine	UPLC	Geometric mean (SE) ug/L BPA: 2.24(0.08) non-NAFLD 2.56(0.15) NAFLD	NAFLD prevalence ALT, AST, GGT	The prevalence of NAFLD and abnormal ALT were increased in accordance with the increase of urinary BPA concentrations. There were no relationships between AST, GGT and BPA levels.	Age, sex, drinking and smoking status, physical activity, household income, education level, marriage, medication taking
BPA	Federico et al. (91)	Italy 2017	Male patients with NAFLD	32	Urine, plasma	HPLC LCMS/MS	mean ± SD ng/mL Plasm BPA: 6.45 ± 4.51 Urine free BPA: 2.73 ± 2.06 Urine total BPA: 5.84 ± 3.07	ALT, AST, GGT	NAFLD patients showed higher levels of ALT, plasmatic, free urine and total urine BPA.	NR
BPA	Kim et al. (92)	USA 2005–2014	NHANES adults	7,605 (HSI) 3,631 (USFLI)	Urine	SPE-HPLC	NAFLD and ALT according to BPA levels.	NAFLD defined by HIS or USFLI	The prevalence of NAFLD and abnormal ALT levels was correlated with urinary BPA levels.	Race/ethnicity, education, hypertension, diabetes, smoking status, alcohol consumption
BPA	Verstraete et al. (93)	Spain 2003–2010	NHANES adolescents (12–19 years)	944	Urine	HPLC-MS	NAFLD and ALT according to BPA levels. Median(IQR) BPA: 2.6(1.3–5.3) ng/mL NAFLD	NAFLD risk	Risk of suspected NAFLD was increased in the second quartile of BPA levels.	Age, gender, race/ethnicity, country of birth, poverty to income ratio, tobacco exposure, daily caloric intake
BPA	Lee et al. (94)	Korea 2005–2016	Children of Ewaha Birth and Growth Cohort Study	164	Urine	HPLC	Median(IQR) ug/L BPA: 0.61(0.35–1.09) 3–5 years old 0.60(0.34–1.15) 7–9 years old	AST, ALT, GGT	The urinary BPA concentrations at 7–9 years was associated with the serum levels of liver enzymes at 10–13 years of age, but 3–5 years not.	sex, age, BMI, monthly household income, maternal educational level, pubertal status, the frequencies of canned fish and soft drink consumption, exposure to secondhand smoke
BPA	Dallio et al. (84)	Italy	NAFLD patients with or without T2DM	60	Urine plasma	LC-MS/MS	Urine BPA: 6.17 ± 0.85 ng/ml NAFLD 0.80 ± 0.17 ng/mL control plasma BPA: 5.30 ± 0.78 ng/ml NAFLD 0.36 ± 0.06 ng/ml control	ALT, AST, GGT grade of NAFLD	BPA resulted to be significantly higher in NAFLD subjects compared to controls both in urine and plasma. BPA plasma levels in NASH patients was higher in NAFL patients.	NR
BPA	Albeldawi et al. (95)	USA 2005–2006	NHANES (18–74 years)	175	Urine	SPE-HPLC-MS/MS	OR (95%CI) Urinary BPA (1 ng/mL, increase): 0.92(0.83, 1.02)	ALT	BPA exposure was not associated with abnormal ALT levels and risk of liver disease.	Age, sex, race/ethnicity, education, smoking, BMI, waist circumference, urinary creatinine concentration

UPLC, ultra-high-performance liquid chromatography; HPLC, high-performance liquid chromatography; SPE, solid-phase extraction; HPLC-MS, high-performance liquid chromatography–tandem mass spectrometry; LC-MS, liquid chromatography-mass spectrometry; LC-MS/MS, liquid chromatography coupled to tandem-mass spectrometry.

### 3.4. Di-(2-ethylhexyl) phthalate

Phthalates are a large group of ubiquitous industrial chemicals that are commonly used in a variety of products such as plasticizers in food wrapping and packaging, coatings, cosmetics, adhesives, medical tubes, and toys (103, 104). Phthalates may enter the human body through the skin, respiratory tract, digestive tract, or even intravenous injection, as they are prone to leaching and transfer to air, soil, or food (105, 106). These chemicals are usually rapidly metabolized and excreted within 24–48 h. Diester phthalates could hydrolyze into monoester phthalates, then excreted as glucuronide conjugates, in the urine (107).

The development of NAFLD may be related to the adverse effects of DEHP on lipid metabolism and oxidative stress. DEHP and its active metabolite mono-(2-ethylhexyl) phthalate (MEPH) can affect hepatic accumulation of TGs and exacerbate NAFLD in rodents (108). MEHP also can affect the lipid accumulation in BRL-3A hepatocytes through the inhibition of the Janus kinase 2/Signal transducer and activator of transcription 5 (JAK2/STAT5) pathway, suggesting that the regulation of STAT5 by MEPH plays a critical role in the activation of enzymes involved in fatty acid metabolism (109). Furthermore, DEHP also mediates the deterioration of antioxidant machinery and induces oxidative stress. Higher levels of ROS were observed in MEHP-treated cells, indicating the effect of ROS on pro-inflammatory cytokine production and apoptosis of hepatocytes by inducing NF-κB (110, 111). Other experimental studies in animals have shown that the toxicity of phthalates drives liver fibrosis by oxidative stress pathways (112, 113).

Phthalate exposure is strongly associated with the NAFLD prevalence (114) (Table 6). A study involving 5,800 Korean adults demonstrated that the prevalence of NAFLD defined by the hepatic steatosis index (HSI) was associated with high urinary levels of many types of phthalates, and higher quartiles of MEHHP revealed a significantly higher risk (OR 1.39, 95% CI:1.00–1.92) of NAFLD (119). In addition, NAFLD measured using vibration-controlled transient elastography (VCTE) was also found to be positively associated with MECPP and MEHHP exposure (116). Unlike in adults, DEHP exposure also affects the prevalence of NAFLD in adolescents. Berman et al. (115) studied 387 mother–child pairs in Australia and found that mid-level prenatal exposure to MnBP was associated with a greater incidence of NAFLD at 17 years old. Table 6 also lists epidemiologic studies on the relationship between NAFLD biomarkers. A study involving 102 males aimed to examine the influence of MEP and MEHP on liver function and found that phthalate exposure may be associated with a statistically significant increase in ALT and AST serum levels, while urinary phthalate levels may be correlated with increased serum TG and decreased HDL cholesterol levels (104). A transversal study also demonstrated that serum MEHP levels were correlated with GGT (122). Thus, DEHP may interfere with thyroid function and induce NAFLD. Yang et al. divided 2,308 adults with subclinical hypothyroidism (SCH) into NAFLD and non-NAFLD groups according to the HSI score and found that the levels of phthalate metabolites in urine are positively associated with NAFLD with SCH (118). DEHP possesses a thyroid receptor antagonistic function, while thyroid hormones can activate TH-Receptor β (a potential target in NAFLD therapy) and decrease hepatic steatosis, which may further induce NAFLD (123–125). However, not all studies have discovered an association between phthalates and thyroid hormones, and further studies ought to be conducted to investigate this association.

**Table 6:** Epidemiologic studies on the relationship between phthalates and NAFLD prevalence and biomarkers.

Substance	Reference	Country	Population	Sample size	Biological materials	Measurement	Exposure assessment	Outcomes	Results	Adjustment factors
DEHP	Berman et al. (115)	Australia 1989–1992 (prenatal)	Mother–child pairs from the Raine Study	387	Maternal serum	LCMS/MS	Phthalate diesters	NAFLD at 17 years old ALT, AST, GGT	Mid-levels of prenatal exposures to MnBP were associated with a greater incidence of NAFLD.	Age, household income at birth, maternal education level at birth, duration of breast feeding, BMI z-score, height
DEHP	Chen et al. (116)	USA 2017–2018	NHANES adults	1,450	Urine	HPLC-ESI-MS/MS	Mean ± SD MECPP: 1.89 ± 0.03 ug/g MEOHP: 0.99 ± 0.03 ug/g MEHHP: 1.44 ± 0.03 ug/g MCiNP: 0.16 ± 0.02 ug/g MCiOP: 1.51 ± 0.04 ug/g MCiNP: 0.19 ± 0.03 ug/g	NAFLD prevalence	Higher prevalence of NAFLD is correlated with MECPP and MEHHP. There is no significant relationship between phthalates and liver fibrosis.	Age, sex, smoking status, education, race/ethnicity, physical activity, diabetes, blood pressure, BMI, total cholesterol levels
DEHP	Fu et al. (90)	China 2018.7–8	Children (5–14 years)	1,006	Serum	HPLC	Median DMP: 31.62 ng/mL	ALT, AST, TBIL	Serum DMP concentration and TBIL level were significantly positively correlated.	Age, address, gender
DEHP	Li et al. (117)	USA 1999–2014	NHANES participants	17,878 (HIS-NAFLD) 8,487 (USFLI-NAFLD)	Urine	HPLC-ESI-MS/MS	13 phthalates OR (95%CI) Urinary phthalates: 1.18(1.09–1.4)	NAFLD prevalence	Urinary phthalates were positively associated with NAFLD development.	Age, sex, race, education, family income-to-poverty ratio, marital status, employment, insurance, self-reported comorbidities, alcohol consumption, cigarettes smoking, leisure time physical activity, diet quality
DEHP	Midya et al. (72)	France, Greece, Lithuania, Norway, Spain, UK 2021–2022	Mother–child pairs from the Human Early-Life Exposome project	1,108	Serum	GC-MS/MS	10 phthalates	ALT, AST, GGT and CK-18 of children	Decreased odds of liver injury were associated with high-molecular-weight phthalates.	Subcohort, maternal age, maternal prepregnancy BMI, maternal educational level, parity, child age, child sex
DEHP	Yang et al. (118)	Korea 2012–2014	Adults with subclinical hypothyroidism from KoNEHS	2,308	Urine	UPLC-MS	Geometric mean(95%CI) ug/L MEHHP: 3.02(2.97–3.06) EH 3.10(2.98–3.23) SCH MEOHP: 2.66(2.61–2.71) EH 2.76(2.64–2.89) SCH MECPP: 3.15(3.10–3.19) EH 3.22(3.11–3.33) SCH MBzP: 1.13(1.05–1.21) EH 1.02(0.84–1.20) SCH MnBP: 3.32(3.26–3.39) EH 3.35(3.22–3.48) SCH	Risk of NAFLD	The levels of phthalate metabolites in urine are positively associated with NAFLD in adults with subclinical hypothyroidism (SCH).	Age, gender, drinking, smoking, physical activity, monthly household income, education, marital status, clinical variables
DEHP	Cai et al. (114)	USA 2003–2016	NHANES adults (>20 years)	4,206	Urine	HPLC-ESI-MS/MS	9 phthalates (MEOHP, MEP, MEHHP, MECPP, MnBP, MEHP, MiBP, MBzP, MCPP)	ALT, AST, GGT	Phthalates exposure was independently associated with NAFLD both in males and females.	Age, gender, education levels, race/ethnicity, marital status, family poverty income ratio, BMI, total cholesterol, survey circle, smoking status, physical activity, hypertension, alcohol consumption
DEHP	Yang et al. (119)	Korea 2012–2014	KoNEHS adults	5,800	Urine	UPLC-MS	GM ± SE MEHHP: 2.922 ± 0.011 ug/L MEOHP: 2.571 ± 0.011 ug/L MECPP: 3.059 ± 0.010 ug/L MnBP: 3.211 ± 0.012 ug/L MBzP: 1.047 ± 0.015 ug/L	NAFLD prevalence	The prevalence of NAFLD was associated with urinary levels of MEHHP, MEOHP, MECPP, MBzP, MnBP compared to the reference group.	Age, gender, smoking, drinking, exercise level, marital status, education level, socioeconomic status.
DEHP	Yu et al. (120)	USA 2007–2016	NHANES adults (≥20 years)	6,046	Urine	HPLC-ESI-MS/MS	15 phthalate metabolites Median ΣDEHP: 3.1 ug/mmol	ALT, AST, ALP, TBIL	Positive dose–response relationships between urinary phthalate metabolites and ALT or AST, ΣDEHP and GGT were observed. Significant positive associations of ΣDEHP with TBIL were found after adjusting for potential confounders.	Age, sex, race/ethnicity, education level, the ratio of family income to poverty, physical activity, alcohol consumption, medications
DEHP	Milošević et al. (121)	Serbia	Adults (18–50 years)	305	Urine	GC-MS	10 phthalates metabolites mean ± SD all phthalates: 304.55 ug/g MEP: 132.2 ± 188.6 ug/g MEHP: 80.36 ± 96.27 ug/g	ALT, AST, GGT	Phthalates exposure was associated with elevated AST levels. ALT and AST values were increased in MEP exposed while GGT levels were enhanced in MEHP exposed.	Obesity, diabetes
DEHP	Milošević et al. (104)	Serbia 2015–2016	Male participants (18–55 years)	102	Urine	GC-MS	MEP, MEHP, MPP, MiAP, MnAP, MCHP, MBzP, MOP, MBP	ALT, AST, GGT	Significant increment in transaminase serum levels was observed in MEP-positive normal weight sub-group. The phthalates exposure may be related to statistically significant ALT and AST serum levels increment.	NR

UPLC, ultra-high-performance liquid chromatography; HPLC, high-performance liquid chromatography; HPLC-MS, high-performance liquid chromatography-tandem mass spectrometry; UPLC-MS, ultra-high-performance liquid chromatography mass spectrometry; LC-MS, liquid chromatography-mass spectrometry; LC-MS/MS, liquid chromatography coupled to tandem-mass spectrometry; HPLC-ESI-MS/MS, High-performance liquid chromatography-electrospray ionization-tandem mass spectrometry; GC-MS, gas chromatograph-mass spectrometry; GC-MS/MS, gas chromatography coupled to tandem mass spectrometry.

### 3.5. Sex differences of association between EDCs and NAFLD

Liver expresses androgen and estrogen receptors (126), thus research on sex-specific differences in EDCs has been a hot topic, but no consistent conclusion has been made so far. In the analyses of NHANES 2013–2016, it was observed that an opposite direction of the statistically significant association between PFAS and liver enzyme by sex, elevated AST is associated with increased PFOA in female adolescents, whereas there is an inverse association with increased PFOA, PFNA and PFHxS in males (54). However, Borghese et al. using the data from Canadian Health Measures Survey found that the association between PFOA and AST was twice as strong among men vs. women, this could be because menstruation, pregnancy, and breastfeeding are all prominent excretion pathways for PFAS in women (25). The sex difference was also reported in PCB, Li et al. (127) found that increased mortality from hepatic disease in PCB-exposed, it may be explained by sex-specific effects of estrogenic PCB congeners (73, 128). Relationships between BPA exposure and liver function at puberty were observed, serum AST levels were positively associated with BPA in boys, and the effect sizes were larger for all indicator in boys (94). Due to the differences in sex hormone associated BPA metabolism, women expressed higher levels of the UGT2B1 to catalyze BPA glucuronidation and accelerate the clearance of BPA (129, 130). Trasande et al. (131) identified a near-significant interaction of DEHP metabolites with sex, suggesting a possible role of reduced androgen activity. EDC’s sex difference is complicated, further research is needed to be done.

## 4. Conclusion

Exposure to environmental chemicals is ubiquitous and poses a threat to human metabolic health. EDCs affect NAFLD by interacting with nuclear receptors (NRs) and activating transcriptional factors, which promote hepatic lipid accumulation, oxidative stress, and liver dysfunction. Data from epidemiological studies prove an interrelationship between EDCs exposure and NAFLD. However, several challenges remain. For example, EDC mixtures are a complicated issue, and it is difficult to predict the net effect of EDC mixtures at the individual level in humans because it is difficult to perform laboratory detection for individual isomer and every human has a unique exposome (22, 90). In addition, the interpretation of the results on the effects of EDCs has been complicated by using different routes of administration under many experimental conditions such as, difference of doses, absence of dose–response relationships, or small sample sizes (132). In the future, research with larger samples, longer follow-up periods, and a multidisciplinary approach to explore the effect of EDCs in the human body is required. Moreover, the scientific community should help draw public attention to the hazards of EDCs and promote more regulation of industrial pollution.

## Author contributions

YC: design, information retrieval, draft writing, and article review and editing. YW: information retrieval, draft writing, and article review and editing. ZC: information retrieval, draft writing, and information visualization. WL: methodology, review, and information visualization. BL: methodology and review. QZ: article review and editing. XZ: article review and editing. JD: methodology, and article review and editing. JC: conceptualization, design, funding acquisition, and article review and editing. All authors contributed to the article and approved the submitted version.

## Conflict of interest

The authors declare that the research was conducted in the absence of any commercial or financial relationships that could be construed as a potential conflict of interest.

## Publisher’s note

All claims expressed in this article are solely those of the authors and do not necessarily represent those of their affiliated organizations, or those of the publisher, the editors and the reviewers. Any product that may be evaluated in this article, or claim that may be made by its manufacturer, is not guaranteed or endorsed by the publisher.
